# Epidermal β-catenin activation remodels the dermis via paracrine signalling to distinct fibroblast lineages

**DOI:** 10.1038/ncomms10537

**Published:** 2016-02-03

**Authors:** Beate M. Lichtenberger, Maria Mastrogiannaki, Fiona M. Watt

**Affiliations:** 1 Centre for Stem Cells and Regenerative Medicine, King's College London, Guy's Hospital, Great Maze Pond, London SE1 9RT, UK; 2 Wellcome Trust Centre for Stem Cell Research, University of Cambridge, Tennis Court Road, Cambridge CB2 1QR, UK

## Abstract

Sustained epidermal Wnt/β-catenin signalling expands the stem cell compartment and induces ectopic hair follicles (EFs). This is accompanied by extensive fibroblast proliferation and extracellular matrix (ECM) remodelling in the underlying dermis. Here we show that epidermal Hedgehog (Hh) and Transforming growth factor-beta (TGF-β) signalling mediate the dermal changes. Pharmacological inhibition or genetic deletion of these pathways prevents β-catenin-induced dermal reprogramming and EF formation. Epidermal Shh stimulates proliferation of the papillary fibroblast lineage, whereas TGF-β2 controls proliferation, differentiation and ECM production by reticular fibroblasts. Hh inhibitors do not affect TGF-β target gene expression in reticular fibroblasts, and TGF-β inhibition does not prevent Hh target gene induction in papillary fibroblasts. However, when Hh signalling is inhibited the reticular dermis does not respond to epidermal β-catenin activation. We conclude that the dermal response to epidermal Wnt/β-catenin signalling depends on distinct fibroblast lineages responding to different paracrine signals.

## Abstract (web summary)

The molecular mechanisms regulating skin dermal changes are unclear. Here, the authors show that deletion of Hedgehog (Hh) in the upper dermis alters the response to epidermal Wnt signalling, which, together with changes in extracellular matrix production, influences distinct fibroblast lineages differently.

## Graphical abstract

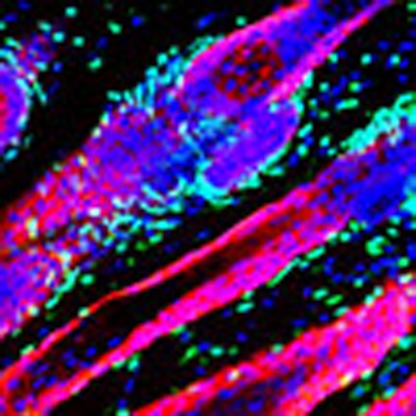

Wnt signalling acts via both cell autonomous and non-cell autonomous mechanisms to regulate skin development and homeostasis^1^. One experimental model that has been used extensively to study the effects of Wnt activation in adult mouse skin is the *K14ΔNβ-cateninER* transgenic mouse^2^. In this model, topical application of 4-hydroxy-tamoxifen (4OHT) leads to expression of N-terminally truncated, constitutively active β-catenin in all epidermal cells that express keratin 14 (K14), including stem cells in different epidermal locations^3^. A single dose of 4OHT is sufficient to induce hair follicles (HFs) in the resting (telogen) phase of the hair growth cycle to enter anagen (growth phase). Sustained Wnt/β-catenin signalling in adult epidermis via repeated doses of 4OHT expands the stem cell compartment and drives cell fate changes, such that cells of the interfollicular epidermis and sebaceous gland form ectopic HFs (EFs)^2^,^4^,^5^.

Epidermal activation of β-catenin not only elicits profound changes within the epidermis itself, but also causes changes in the underlying connective tissue, characterized by increased fibroblast proliferation and extensive remodelling of the dermal extracellular matrix (ECM)^6^. Recently, the fibroblasts of the upper, papillary, dermis have been shown to originate from a different lineage to those of the lower, reticular dermis and dermal adipocytes^7^. The papillary lineage is required for HF formation in skin reconstitution assays, whereas the reticular lineage produces the bulk of the ECM and is responsible for the first wave of dermal repair following a full thickness wound. Epidermal Wnt activation in *K14ΔNβ-cateninER* mice leads to an increase in the abundance of both papillary and reticular lineages and as a result new HFs form in the epidermal wound bed^4^,^7^.

In the present study, we set out to identify the signalling mechanisms by which epidermal Wnt activation remodels the dermis and to determine whether the papillary and reticular dermal fibroblasts respond to the same or different signals. We find that on Wnt/β-catenin activation, the epidermis expresses Sonic hedgehog (Shh), which stimulates proliferation and ECM remodelling by the papillary dermis, whereas the reticular dermis responds to epidermal Transforming growth factor (TGF)-β. These findings are of particular interest, given the many different epithelial tumours in which there is inappropriate activation of Wnt signalling accompanied by changes in the underlying connective tissue^8^,^9^,^10^.

## Results

### Epidermal β-catenin causes intrinsic fibroblast changes

**Figure 1:**
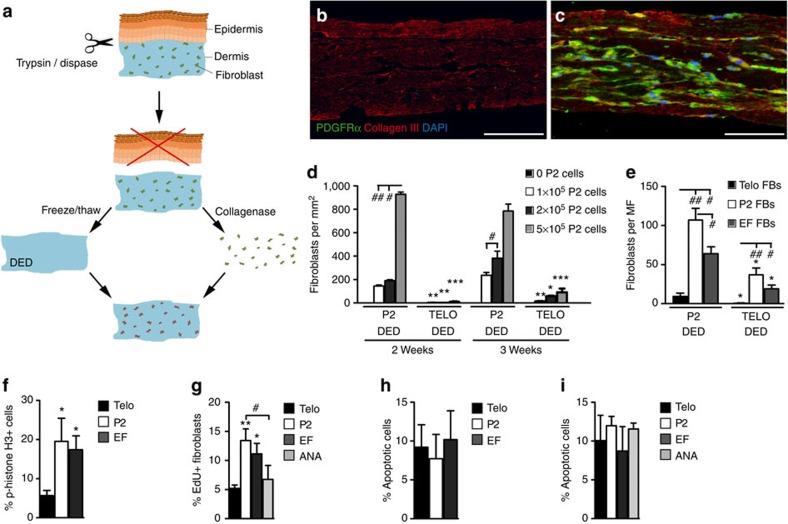
Reprogrammed fibroblasts retain increased proliferative potential in culture. (**a**) Outline of experimental procedure for preparing and repopulating de-epidermized dermis (DED) from murine skin. (**b**,**c**) Sections of P2 DEDs after 2 weeks of culture stained with antibodies to PDGFRα (green) and collagen 3 (red), counterstained with 4,6-diamidino-2-phenylindole (DAPI; blue). DEDs were unseeded (**b**) or seeded with 2 × 10^5^ neonatal fibroblasts (**c**). Scale bars, 50 μm (**d**) Quantification of fibroblasts isolated from 2-day-old mice and seeded onto P2 or adult telogen (TELO) DEDs. Fibroblasts were cultured in DMEM/10% FCS for 2–3 weeks. *P≤0.05; **P≤0.005; ***P≤0.0005 between cells seeded on neonatal or adult DEDs; ^#^*P*≤0.05; ^##^*P*≤0.005; compared with 1 × 10^5^ seeded fibroblasts; by a one-way analysis of variance (ANOVA). (**e**) Quantification of fibroblasts isolated from neonatal (P2), telogen skin (Telo) or skin containing ectopic follicles (EF). (**e**) *P≤0.05; between cells seeded on neonatal or adult DEDs; ^#^*P*≤0.05; ^##^*P*≤0.005; compared with telogen; by a one-way ANOVA. MF, microscopic field. (**d**,**e**) Data show means±s.e.m. of triplicate samples in a representative experiment (*n*=3 independent experiments). (**f**-**i**) Bar graphs showing percentage of proliferating (**f**,**g**) and apoptotic (**h**,**i**) fibroblasts isolated from neonatal (P2), telogen (Telo), anagen (ANA) and reprogrammed (EF) skin quantified 48 h after seeding flow-sorted fibroblasts onto culture dishes (**g**,**i**) or 3 weeks after seeding flow-sorted fibroblasts onto P2 DEDs (**f**,**h**). (**f**–**i**) Data represent mean±s.e.m. (*n*=3). *P≤0.05, **P≤0.005 by a one-way ANOVA, compared with telogen; ^#^*P*≤0.05, compared with P2.

To address whether the stimulation of fibroblast proliferation in response to epidermal Wnt/β-catenin activation is a cell intrinsic effect or a response to changes in the dermal ECM, we developed a dermal reconstitution assay. The epidermis was enzymatically removed from skin biopsies of neonatal (P2) or adult (telogen; resting phase of the hair growth cycle) back skin and the dermis was de-vitalized through repeated freeze/thaw cycles (Fig. 1a). The resulting de-epidermized dermis (DED) was placed on a cell culture insert, seeded with fibroblasts isolated directly from P2 skin and cultured for 2–3 weeks. By 2 weeks, the fibroblasts had colonized the full thickness of the dermis, as visualized by labelling for the pan-fibroblast marker, Platelet-derived growth factor receptor alpha (Pdgfrα) (Fig. 1b,c). Fibroblasts isolated from neonatal skin expanded more extensively in neonatal than adult telogen DED at all three seeding densities and both time points tested (Fig. 1d), demonstrating that the dermal ECM had an impact on fibroblast proliferation.

We next examined the effect of epidermal Wnt/β-catenin activation by applying 4OHT to the back skin of telogen *K14ΔNβ-cateninER* mice^2^ to induce EFs. We then compared the proliferation of fibroblasts from untreated telogen skin, wild-type P2 skin and skin containing EFs (Fig. 1e). Telogen fibroblasts showed limited proliferation in either P2 or telogen DEDs. Fibroblasts isolated from the skin with EFs were more proliferative than telogen fibroblasts and, like P2 fibroblasts, proliferated more extensively in P2 DEDs than telogen DEDs (Fig. 1e,f). This was also the case for fibroblasts cultured on tissue culture plastic (Fig. 1g). In contrast, the proportion of apoptotic fibroblasts did not differ significantly between wild-type P2, adult telogen and anagen skin or 4OHT-treated *K14ΔNβ-cateninER* skin (Fig. 1h,i). We conclude that fibroblast proliferation is influenced both by the dermal ECM (Fig. 1d,e) and by cell-intrinsic changes resulting from postnatal age and epidermal Wnt-activation (Fig. 1e–g).

### Sequential induction of epidermal growth factors (GFs)

**Figure 2:**
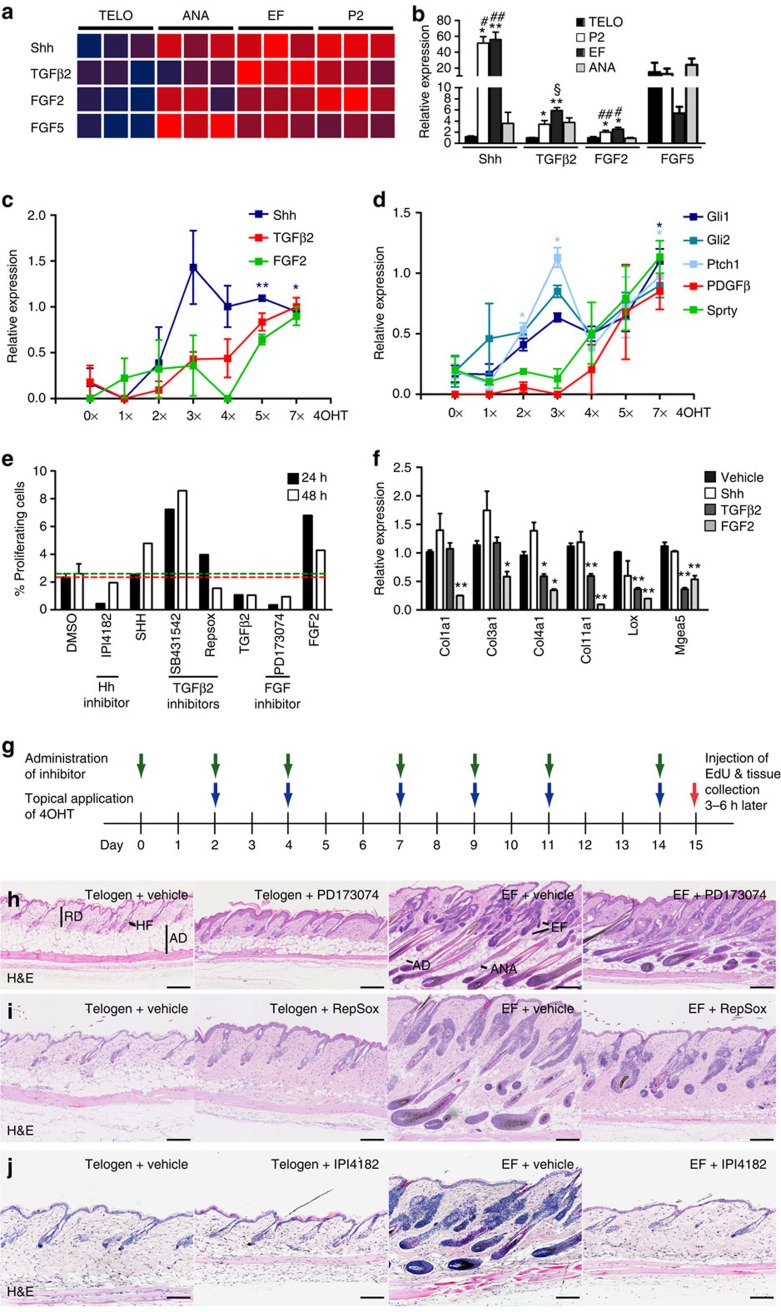
Pharmacological inhibition of FGF, TGF-β or Hh signalling impairs ectopic follicle (EF) formation *in vivo*. (**a**) Heat map (blue: low expression; red: high expression) showing differential expression of growth factors in epidermis from neonatal, adult telogen (resting phase), anagen (active phase) and reprogrammed (EF) skin. (**b**) Relative expression of the indicated growth factors in flow-sorted epidermal cells measured by qRT–PCR. Data represent mean±s.e.m. (*n*=3–4). *P≤0.05; **P≤0.005; compared to telogen; ^#^*P*≤0.05; ^##^*P*≤0.005; compared to anagen; ^§^*P*≤0.05; compared to P2; by a one-way analysis of variance (ANOVA). **c**,**d**) Relative expression of growth factors in epidermal cells (**c**) and growth factor target genes in fibroblasts (**d**) isolated from *K14ΔNβ-cateninER* transgenic mice treated seven times with vehicle alone (0x) or with 4OHT once or up to seven times. Data represent mean±s.e.m. (*n*=2). *P≤0.05; **P≤0.005; by Student's *T* test. (**e**) Percentage of proliferating (EdU+) fibroblasts 24 or 48 h after treatment with the indicated growth factors or inhibitors. Green and red dotted lines represent the mean percentage of vehicle-treated cells after 24 h (red) and 48 h (green). Cells were pulsed with EdU for 2 h. Cells were pooled from three technical replicates and the results are representative of *n*=3 independent experiments. (**f**) Bar graphs showing relative expression of the indicated genes in fibroblasts isolated from adult skin and treated with Shh, TGF-β2, FGF2 or vehicle *in vitro*. Data represent mean±s.e.m. (*n*=3). *P≤0.05; **P≤0.005 by a one-way analysis of variance. (**g**) Treatment scheme. Topical application of 4OHT induces Wnt/β-catenin activation in the epidermis of *K14ΔNβ-cateninER* transgenic mice. Inhibitors were applied by subcutaneous injection (RepSox, PD173074) or oral gavage (IPI4182) every other day. (**h**–**j**) Inhibition of FGF (**h**), TGF-β (**i**) Hh (**j**) signalling impairs or blocks induction of anagen and EFs in *K14ΔNβ-cateninER* transgenic mice. Representative haematoxylin and eosin stainings are shown. AD, adipocytes; ANA, anagen hair follicle; HF, hair follicle; RD, reticular dermis. Scale bars, 200 μm.

To identify candidate epidermal secreted factors that mediate the dermal changes, we analysed published microarray data from neonatal, telogen and anagen (growth phase of hair cycle) epidermis, and epidermis that had formed EF in response to β-catenin activation (three biological replicates per condition)^6^. Secreted GFs found to be upregulated in neonatal, anagen and EF epidermis but not telogen epidermis included Shh, TGF-β2, Fibroblast growth factors 2 (FGF2) and 5 (FGF5) (Fig. 2a). Quantitative reverse transcription–PCR (qRT–PCR) confirmed that Shh, TGF-β2 and FGF2 were highly expressed in neonatal and EF epidermal cells compared with keratinocytes from telogen skin (Fig. 2b). Expression of Shh, FGF2 and TGF-β2 was higher in P2 and EF epidermis than in anagen epidermis. In contrast, FGF5 expression was lower in EF keratinocytes than the other keratinocyte populations (Fig. 2b).

To examine the kinetics of GF signalling *in vivo*, we treated *K14ΔNβ-cateninER PDGFRαH2BeGFP* (expressing eGFP in nuclei of all fibroblasts)^6^ double transgenic mice with 4OHT (or vehicle) once or every other day for up to a total of seven applications. Twenty-four hours after the last treatment, the epidermis was separated from the dermis. Basal layer keratinocytes were flow-sorted from the epidermis on the basis of Integrin α6 (ITGA6) expression, and total dermal fibroblasts on the basis of expression of PDGFRαH2BeGFP. As reported previously^2^, two doses of 4OHT were sufficient to induce anagen, whereas after five treatments there was pronounced EF formation (Supplementary Fig. 1). Activated (non-phosphorylated) β-catenin was detected in all transgene-positive regions of the epidermis following 4OHT treatment (Supplementary Fig. 1).

QRT–PCR analysis of mRNA from flow-sorted epidermal cells showed that *Shh* expression peaked after three 4OHT treatments, whereas *TGF-β2* and *FGF2* levels rose later (Fig. 2c). Consistent with those kinetics, the Hh target genes *Gli1, Gli2* and *Patched 1* (*Ptch1*) were maximally expressed in fibroblasts after three 4OHT treatments, whereas levels of TGF-β and FGF target genes—*Pdgfβ* and *Sprouty*, respectively—increased after four treatments (Fig. 2d).

Based on previous reports^11^,^12^,^13^,^14^, we predicted that the effects of Shh and FGF2 would be to stimulate fibroblast proliferation, whereas TGF-β2 should inhibit proliferation and promote ECM remodelling. Treatment of adult fibroblasts with FGF2 and Shh induced proliferation in culture, whereas, as reported previously^15^, TGF-β2 was inhibitory (Fig. 2e). Accordingly, the FGFR inhibitor PD173074 (Tocris) and the Hh/Smoothened inhibitor IPI4182 (ref. 16; Infinity Pharmaceuticals; compound 32) blocked fibroblast proliferation, whereas two TGF-β inhibitors (RepSox, Sigma Aldrich; and SB431542, Tocris) stimulated proliferation (Fig. 2e). In cultured fibroblasts, TGF-β2 and FGF2 significantly reduced expression levels of a range of collagen genes and also of *lysyl oxidase* (*Lox*), which cross-links collagen and elastin^17^, and the hyaluronidase *Mgea5* (Fig. 2f). In contrast, Shh did not affect the expression of collagens and ECM-modifying enzymes (Fig. 2f). These results suggest that the increase in epidermal Shh and FGF2 expression upon β-catenin activation drives the increase in fibroblast proliferation, whereas the increases in TGF-β2 and FGF2 mediate the changes in ECM.

### Effects of GF inhibition

To examine the roles of the candidate secreted factors *in vivo*, *K14ΔNβ-cateninER PDGFRαH2BeGFP* double transgenic mice and *PDGFRαH2BeGFP* control littermates were treated with PD173074, RepSox or IPI4182. We compared telogen mice (4OHT-treated *PDGFRαH2BeGFP* single transgenics) with *K14ΔNβ-cateninER PDGFRαH2BeGFP* double transgenic mice treated with 4OHT to induce ectopic follicle formation for 2 weeks (Fig. 2g).

Haematoxylin and eosin (H&E) staining revealed that all three inhibitors interfered with the epidermal and dermal responses to β-catenin activation. The FGFR inhibitor had the mildest effect: HF still entered anagen and formed EFs, but follicle length was reduced (Fig. 2h; *n*=8). In addition, telogen skin treated with the FGFR inhibitor displayed epidermal hyperproliferation but no changes in the dermis (Fig. 2h; *n*=8). In the presence of RepSox, anagen and EF formation was largely inhibited (Fig. 2i; *n*=10–11). RepSox treatment also resulted in an increased fibroblast density and reduction in the skin adipocyte layer, whether applied to wild-type telogen or *K14ΔNβ-cateninER* transgenic 4OHT-treated skin (Fig. 2i; *n*=10–11 per condition). The Hh inhibitor had no effect on telogen skin (*n*=10), but the strongest effect of the three inhibitors on *K14ΔNβ-cateninER* transgenic 4OHT-treated skin, resulting in complete inhibition of Wnt-mediated anagen and EF induction (9 out of 12 mice; Fig. 2j).

**Figure 3:**
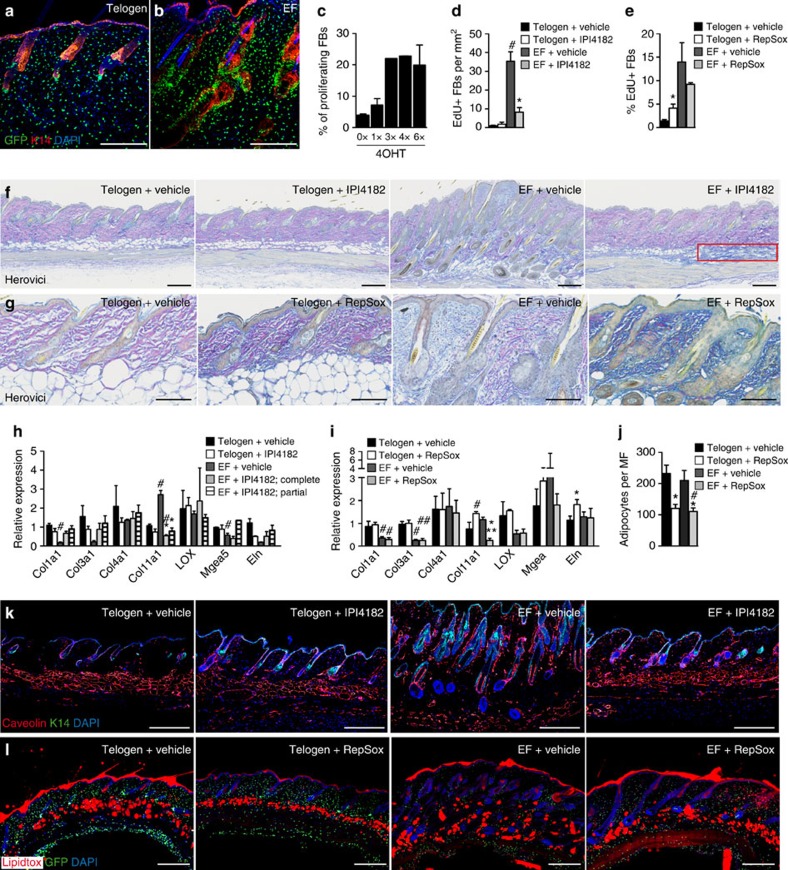
Effects of pharmacological Hh and TGF-β inhibitors on fibroblast proliferation, ECM remodelling and adipogenesis. (**a**,**b**) Immunofluorescent staining of skin sections from telogen and reprogrammed skin from *K14ΔNβ-cateninER/ PDGFRαH2BeGFP* double transgenic mice showing an increase in dermal fibroblasts upon epidermal Wnt/β-catenin activation. Keratin 14 is expressed in epidermal cells. All fibroblasts express H2BeGFP in their nuclei. Nuclei were detected with 4,6-diamidino-2-phenylindole (DAPI). (**c**–**e**) Quantification of proliferating fibroblasts in *K14ΔNβ-cateninER/ PDGFRαH2BeGFP* double transgenic mice treated with up to six doses of 4OHT (**c**) or treated with Hh (**d**) and TGF-β inhibitors (**e**). Quantification was performed by FACS (**c**,**e**) or in immunolabelled skin sections (**d**). Mice were injected with EdU 3 (**c**,**e**) or 4 h (**d**) before tissue collection. Data represent mean±s.e.m. (*n*=3–4). *P≤0.05, inhibitor-treated compared with vehicle-treated mice; ^#^*P*≤0.05, EF skin compared with telogen skin, by a one-way analysis of variance (ANOVA). (**f**,**g**) Herovici staining of skin sections from telogen and reprogrammed (EF) skin treated with Hh (**f**) or TGF-β inhibitors (**g**). Immature collagen fibres appear blue, whereas mature collagen is stained pink. Red box highlights a patch of immature collagen in the lower dermis (**f**). (**h**,**i**) Relative expression of the indicated genes in fibroblasts isolated from telogen and reprogrammed (EF) skin treated with IPI4182, RepSox or vehicle. Data represent mean±s.e.m. (*n*=4–5). (**j**) Number of adipocytes per microscopic field (MF) quantified in H&E-stained sections. Data represent mean±s.e.m. (*n*=3). (**h**–**j**) *P≤0.05; **P≤0.005, inhibitor-treated compared with vehicle-treated mice; ^#^*P*≤0.05, ^##^*P*≤0.005 compared with telogen skin by a one-way ANOVA. (**k**,**l**) Immunofluorescent staining of skin sections with antibodies to Caveolin and K14 or Lipidtox, counterstained with DAPI. Scale bars in **a**,**b**,**f**,**g**,**k** and **l** represent 200 μm.

Since the effects of Hh and TGF-β inhibition were the most pronounced, we examined the changes in the dermis in more detail. As previously reported^7^,^18^ there was an increase in the number of fibroblasts in all regions of the dermis in response to epidermal Wnt/β-catenin activation (Figs 2h–j and 3a,b) and an increase in the thickness of the adipocyte layer^18^ (Fig. 2h–j). 5-Ethynyl-2′-deoxyuridine (EdU) labelling demonstrated an increase in fibroblast proliferation, which was maximal after three doses of 4OHT (Fig. 3c). Treatment with IPI4182 significantly reduced the proliferative response to 4OHT (Fig. 3d). In contrast, RepSox treatment increased fibroblast proliferation in telogen skin (Fig. 3e), consistent with the increased fibroblast density observed in H&E-stained RepSox-treated telogen skin sections (Fig. 2i). RepSox treatment did not, however, have a statistically significant effect on fibroblast proliferation in 4OHT-treated *K14ΔNβ-cateninER* transgenic skin (Fig. 3e).

To investigate whether the inhibitors affected epidermal β-catenin-induced dermal ECM remodelling, we performed Herovici staining (Fig. 3f,g). This stain labels mature fibrillar collagen pink and immature collagen blue^19^. Both IPI4182-treated and untreated telogen dermis had a preponderance of mature collagen, whereas untreated dermis of mice with epidermal β-catenin activation had a high proportion of immature collagen, as reported previously^6^ (Fig. 3f). Skin in which EF formation was blocked with IPI4182 was similar to telogen skin but displayed patches of immature collagen in the lower dermis (Fig. 3f). In contrast, inhibition of TGF-β in 4OHT-treated *K14ΔNβ-cateninER* skin resulted in extensive ECM remodelling as shown by intense blue Herovici staining (Fig. 3g).

Confirming the histological analysis, the effects of RepSox on expression of several collagen genes, *elastin* and ECM-modifying enzymes were more pronounced than those of IPI4182 (Fig. 3h,i). However, Collagen type 11, which is upregulated in fibroblasts in close proximity to EFs^6^, was inhibited by IPI4182, RepSox and the FGFR inhibitor PD173074 (Fig. 3h,i and Supplementary Fig. 2a). Immunofluorescent staining for Col3a1, Col11a1 and Elastin (Supplementary Fig. 2b–e) was consistent with the Herovici labelling and qRT–PCR data (Fig. 3f–i).

In addition to examining the composition of the dermal ECM, we determined the thickness of the skin adipocyte layer by labelling mature adipocytes with anti-caveolin or Lipidtox (Fig. 3j–l). Treatment of telogen skin with the Hh inhibitor did not affect the thickness of the skin adipocyte layer, but prevented the increase in adipose layer thickness that normally occurs on epidermal β-catenin activation (Fig. 3k). In contrast, RepSox significantly reduced the number of mature adipocytes in telogen and 4OHT-treated *K14ΔNβ-cateninER* skin (Fig. 3j,l).

To confirm that the inhibitors were indeed targeting the appropriate pathways, we performed qRT–PCR of fibroblasts isolated directly from mouse skin (Supplementary Fig. 3). Comparison of IPI4182- and vehicle-treated back skin showed that expression of the Hh target genes *Gli1*, *Gli2* and *Ptch1* was significantly downregulated when there was a complete block in EF formation. Moreover, in those mice that had only partial inhibition of the β-catenin-induced phenotype (3 out of 12 mice) there was no reduction in target gene expression (Supplementary Fig. 3a). Levels of TGF-β (*Id1, Id2, Myc, Cxcl12*) and FGF (*Sprouty*) target genes were not affected by the Hh inhibitor (Supplementary Fig. 3b). *Gli1, Gli2, Ptch1* and *Shh* expression levels were not affected in epidermal cells isolated from inhibitor-treated skin (Supplementary Fig. 3c), indicating that inhibition of EF formation was due to an effect on fibroblasts rather than epidermal cells.

RepSox reduced expression of the TGF-β target gene *Id1*, whereas expression of Hh and FGF target genes was not affected (Supplementary Fig. 3d). The FGFR inhibitor enhanced expression of Hh target genes in telogen skin and of *Id1* in 4OHT-treated *K14ΔNβ-cateninER* skin (Supplementary Fig. 3e). In accordance with the mild effect on EF formation, the FGFR inhibitor did not effectively inhibit expression of the FGF target gene *Sprouty*, which was also not significantly affected by RepSox and IPI4182 (Supplementary Fig. 3b,d,e). These data suggest that there is no cross-talk between the Hh and TGF-β signalling pathways but that FGFR signalling affects both pathways.

### Effects of GF inhibition on fibroblast subpopulations

**Figure 4:**
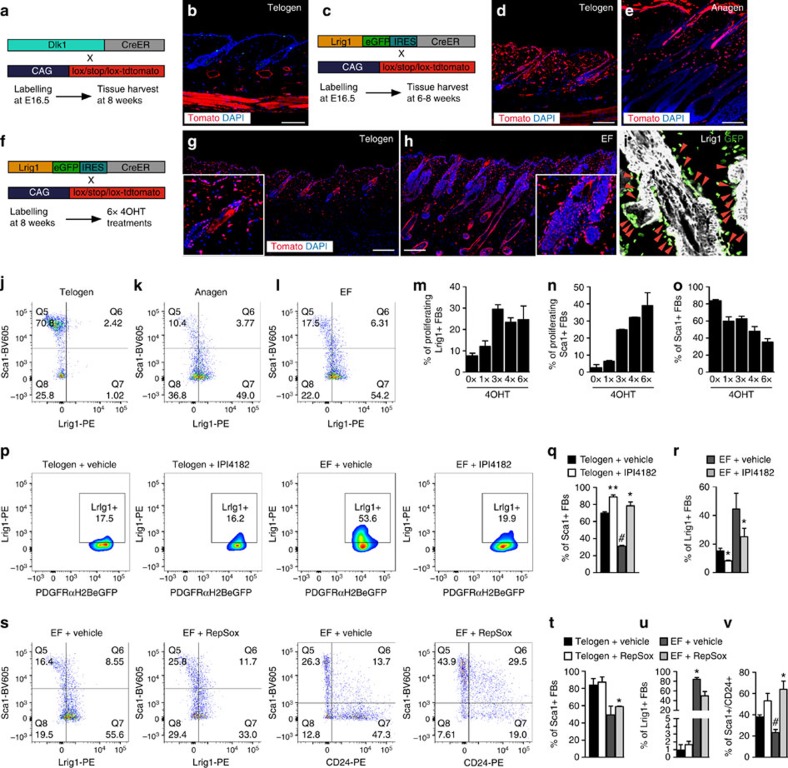
Hh and TGF-β inhibitors affect different fibroblast lineages. (**a**,**b**) Labelling of the reticular dermal lineage using Dlk1 expression. (**a**) Experimental strategy; (**b**) adult telogen skin. (**c**–**e**) Labelling the papillary dermal lineage using Lrig1 expression. (**c**) Experimental strategy; (**d**,**e**) adult telogen (**d**) and anagen (**e**) skin. (**f**–**h**) Labelling Lrig1+ cells in adult skin. (**f**) Experimental strategy; (**g**,**h**) *K14ΔNβ-cateninER* (**h**) and wild-type littermate control (**g**) skin following six doses of 4OHT. (**b**,**d**,**e**,**g**,**h**) Sections were stained for tdTomato and nuclei were detected with 4,6-diamidino-2-phenylindole (DAPI). Scale bars represent 200 μm (**b**,**d**,**e**) and 100 μm (**g**,**h**). (**i**) Section of 4OHT-treated *K14ΔNβ-cateninER* skin stained for Lrig1 and GFP. Red arrow heads show Lrig1+ fibroblasts associated with ectopic HF. (**j**–**l**) Flow cytometry plots showing fibroblast populations isolated from telogen (**j**), anagen (**k**) and EF (**l**) skin. PDGFRαeGFP was used to gate on fibroblasts. Plots are representative of *n*=3-4 mice per condition. (**m**–**o**) Bar graphs showing flow cytometric quantification of proliferating Lrig1+ and Sca1+ (**m**,**n**) and total Sca1+ fibroblasts (**o**). Data represent mean±s.e.m. (*n*=3–4). (**m**,**n**) Cells were labelled for 4 h with EdU. (**p**–**v**) Representative flow cytometry plots (**p**,**s**) and summary of flow cytometric analysis of fibroblast populations after gating on PDGFRαH2BeGFP+ cells (**q**,**r**,**t**–**v**) isolated from telogen and reprogrammed (EF) skin treated with the Hh inhibitor IPI4182 (**p**–**r**), RepSox (**s**–**v**) or vehicle. Data represent mean±s.e.m. (*n*=3–4). *^,#^*P*≤0.05; **P≤0.005 by a one-way analysis of variance, compared with telogen or EF skin, respectively.

To examine the effects of epidermal β-catenin activation on papillary and reticular dermal fibroblasts, we sought to identify markers that would allow us to distinguish them. We have previously shown that at E16.5 dermal fibroblasts that will give rise to the upper dermal lineages (papillary fibroblasts, including cells of the dermal sheath (DS), dermal papilla (DP) and arrector pili muscle) express leucine-rich repeats and immunoglobulin-like domains protein 1 (Lrig1), whereas cells that will give rise to the lower dermis and adipocyte layer express Delta-like 1 (Dlk1)^7^. Lineage tracing using *Dlk1-CreER*^7^ or *Lrig1-GFP-IRES-CreER*^20^ (*Lrig1CreER*) *Rosa-CAG-loxP-stop-loxP*^21^ (hereafter *LSL-tdTomato* mice, in which tdTomato is expressed when the stop codon is removed via Cre-mediated recombination) mice injected with tamoxifen at E16.5 confirmed that in adult telogen mice (8 weeks) the Dlk1 lineage was confined to the lower dermis (Fig. 4a,b). Dlk1 protein is not expressed in postnatal fibroblasts^7^, but Sca1 can be used as a positive marker of lower dermal fibroblasts, including adipocytes^7^.

When Lrig1+ cells are marked with a low labelling efficiency at E16.5, the majority of postnatal tdTomato+ cells are in the arrector pili muscle (APM) and DP^7^. However, more efficient induction of *Lrig1CreER* at E16.5 results in more extensive labelling of dermal fibroblasts. In addition to accumulation of labelled cells in the dermal sheath, there was also labelling in the reticular layer (Fig. 4c–e), and we detected occasional tdTomato+/Sca1+ cells in the adipocyte layer. When *Lrig1CreER x LSL-tdTomato* mice received multiple applications of 4OHT, beginning at 8 weeks, the same distribution of labelled fibroblasts is found (Fig. 4f,g). Induction of ectopic follicles in 4OHT-treated *K14ΔNβ-cateninER* skin resulted in a marked increase in Lrig1+ fibroblasts, with large numbers confined to the dermal sheath (Fig. 4h,i), whether detected by antibody labelling (Fig. 4i) or 4OHT-mediated tdTomato labelling (Fig. 4f,h).

We also examined the expression of CD26 (dipeptidyl peptidase-4), which is restricted to papillary fibroblasts and DP cells in neonatal skin, but expressed by the majority of reticular fibroblasts in adult skin^7^,^22^,^23^ (Supplementary Fig. 4a,b). DP cells are CD26+ in adult telogen but not anagen follicles (Supplementary Fig. 4a). Flow cytometric analysis of adult skin fibroblasts revealed that in telogen the majority of fibroblasts (75–85%) were Sca1+/CD26+ (Supplementary Fig. 4b) and only a few cells were Lrig1+ (Fig. 4j). In contrast, anagen skin harboured a small percentage of Sca1+/CD26+ cells, whereas almost 50% of cells were Lrig1+/Sca1− (Fig. 4k). Anagen skin also contained a large number of Sca1−/CD26− fibroblasts, which included DP cells expressing CD133 (ref. 24; 20–25%) and Lrig1+ cells (45–60%; Supplementary Fig. 4b).

Induction of ectopic follicles in 4OHT-treated *K14ΔNβ-cateninER* skin resulted in a further increase in Lrig1+cells (55–70%) and Sca1−/CD26− cells compared with anagen skin, with most of the Sca1−/CD26− cells expressing Lrig1 or CD133, and <25% Sca1+ fibroblasts (Fig. 4l,p and Supplementary Fig. 4d). Although both Lrig1+ and Sca1+ fibroblasts proliferated in response to multiple applications of 4OHT (EdU+; Fig. 4m,n), the ratio of Lrig1+ to Sca1+ fibroblasts increased dramatically (from 0.19 to 2.6; Fig. 4o).

The Hh inhibitor IPI4182 blocked the β-catenin-induced increase in Lrig1+ fibroblasts (Fig. 4p,r) and therefore the increase in Sca1−/CD26− cells. As a result, the proportion of Sca1+ fibroblasts was restored to the level found in telogen skin (Fig. 4q). RepSox treatment had little effect on the proportion of Lrig1+ and Sca1+ fibroblasts in telogen skin but reduced the proportion of Lrig1+ (and also Sca1−/CD26−) fibroblasts and increased the proportion of Sca1+ fibroblasts on activation of β-catenin (Fig. 4s–u). The reduction in mature adipocytes on RepSox treatment (Fig. 3j) correlated with an increase in Sca1+/CD24+ pre-adipocytes (Fig. 4s,v).

These results show that although blocking either Hh or TGF-β inhibits β-catenin-induced EF formation, the inhibitors act on different fibroblast subpopulations. Hh inhibition primarily affects Lrig1+ fibroblasts, whereas TGF-β inhibition exerts its effect on lower dermal fibroblasts and pre-adipocytes.

### *Smoothened* deletion in fibroblasts blocks dermal responses

Hh signalling has been shown to be important for HF morphogenesis and HF cycling^25^ and epidermal deletion of the Hh effector gene *Smoothened* results in loss of HFs and hyperproliferation of the interfollicular epidermis (Supplementary Fig. 5a,b). To confirm that the effects on EF formation of treatment with the Hh inhibitor IPI4182 were indeed due to a response of the fibroblasts and not the epidermal cells, we crossed our mouse model to conditional *Smoothened* mice (*Smof/f*)^26^ and a *Dermo1Cre* (*B6.129X1-Twist2*^*tm1.1(cre)Dor*^*/J*)^27^ transgenic line, enabling us to delete Smoothened specifically in fibroblasts (Supplementary Fig. 5c–e). Deletion of both Smoothened alleles by this Cre line was embryonic lethal, but heterozygous mice exhibited a normal lifespan. The heterozygous mutants had significantly fewer HF compared with wild-type skin (83.39%±0.03%; *n*=12; *P*=0.0011) and entry into the anagen phase of the hair growth cycle became desynchronized after the first postnatal hair cycle.

**Figure 5:**
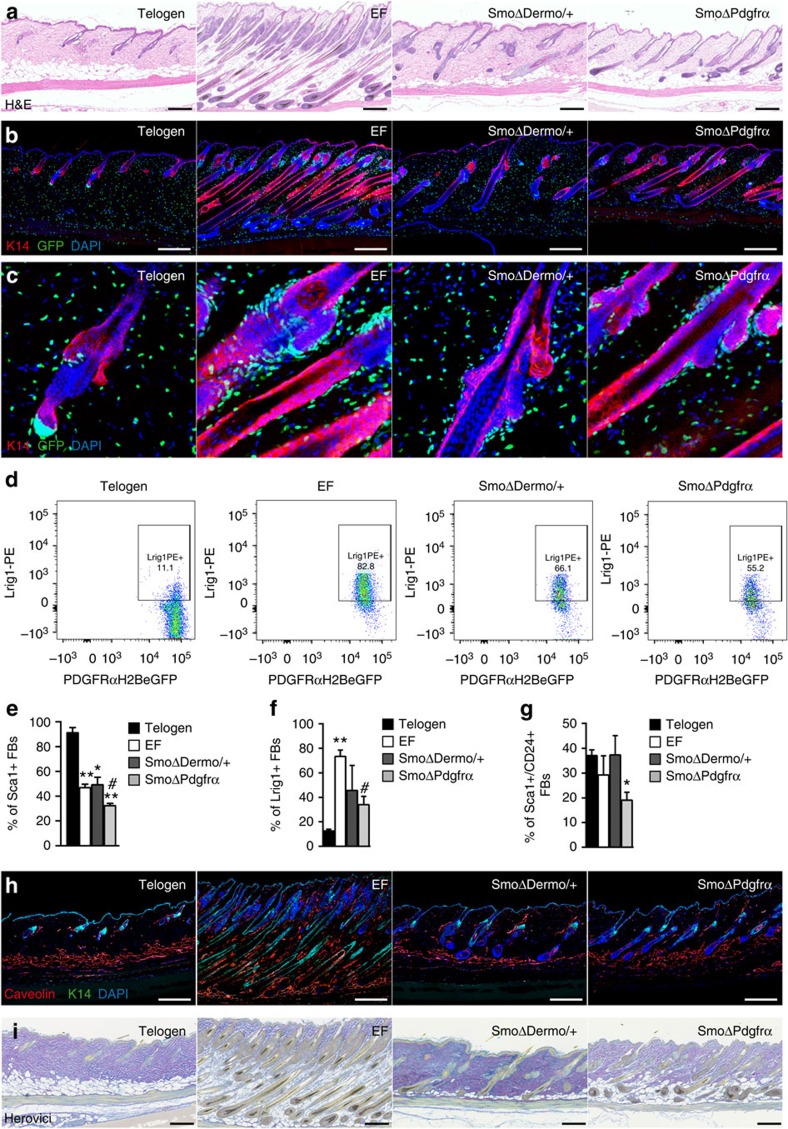
Deletion of *Smoothened* in fibroblasts affects EF formation. *K14ΔNβ-cateninER* mice were examined in telogen or following 4OHT treatment to induce ectopic hair follicles (EFs) in combination with heterozygous *Smoothened* deletion via *Dermo1Cre* (*SmoΔDermo/+*) or homozygous *Smoothened* deletion via *PdgfraCreER* (*SmoΔPdgfrα*). (**a**–**c**) H&E (**a**) and immunofluorescent staining (**b**,**c**) of dorsal skin sections. In **b**,**c**, sections were labelled with antibodies detecting GFP and K14. 4,6-Diamidino-2-phenylindole (DAPI) was used to visualize nuclei. (**c**) Higher magnification images of **b**. (**d**) Representative flow cytometry plots of Lrig1+ fibroblasts after gating on PDGFRαH2BeGFP+ cells. (**e**–**g**) Summary of flow cytometric analysis. Data represent mean±s.e.m. (*n*=3). *^,#^*P*≤0.05; **P≤0.005 by a one-way analysis of variance, compared with telogen or EF skin, respectively. (**h**) Immunofluorescent staining with antibodies detecting Caveolin and K14, counterstained with DAPI. (**i**) Herovici staining. Scale bars, 200 μm.

Deletion of a single *Smoothened* allele (*SmoΔDermo/+ mice*) significantly reduced EF formation (Fig. 5a). *SmoΔDermo/+* skin harboured fewer fibroblasts throughout the papillary and reticular dermis and in the dermal sheath (Fig. 5b,c and Supplementary Fig. 5f), and displayed a shift towards the telogen phenotype in the proportions of Lrig1+, Sca1+ and Sca1+/CD24+ fibroblasts (Fig. 5d-g), with fewer Lrig1+ fibroblasts than EF skin. In *K14ΔNβ-cateninER SmoΔDermo/+* mice, the number of Lrig1+ fibroblasts accumulating around HF after three 4OHT treatments was much lower than the number in *K14ΔNβ-cateninER Smof/f* mice (Supplementary Fig. 5g, h). Although the number of pre-adipocytes was not affected by *Smo* deletion (Fig. 5g), *K14ΔNβ-cateninER SmoΔDermo/+* skin harboured fewer mature adipocytes (Fig. 5h and Supplementary Fig. 4i), and ECM remodelling was reduced in *K14ΔNβ-cateninER SmoΔDermo/+* mice (Fig. 5i). The expression of collagens was also altered compared with telogen skin as assessed by Herovici staining (Fig. 5i) but not by qRT–PCR analysis of several ECM genes (Supplementary Fig. 5j).

To confirm that preconditioning the dermis during development was not required to impair EF formation, we also deleted *Smoothened* in adult mice via *PDGFRα-CreER*^28^ (Supplementary Fig. 5k, l). 4OHT treatment simultaneously deleted *Smoothened* in the dermis (*SmoΔPdgfrα*) while activating β-catenin in the epidermis. This led to inhibition of EF formation and dermal reprogramming (Fig. 5a–i). In fibroblasts isolated from *SmoΔPdgfrα* skin, expression of Hh target genes was significantly reduced compared with EF fibroblasts with wild-type *Smoothened* and comparable to telogen levels (Supplementary Fig. 5m). In contrast, expression levels of *Gli1*, *Gli2* and *Ptch1* in *SmoΔDermo/+* fibroblasts were similar to EF fibroblasts with wild-type *Smoothened*, consistent with the retention of one Smoothened allele (Supplementary Fig. 5m). These studies demonstrate that epidermal β-catenin-mediated EF formation is dependent on Hh responsiveness of dermal fibroblasts.

### Hh and TGF-β inhibition affect distinct fibroblast lineages

**Figure 6:**
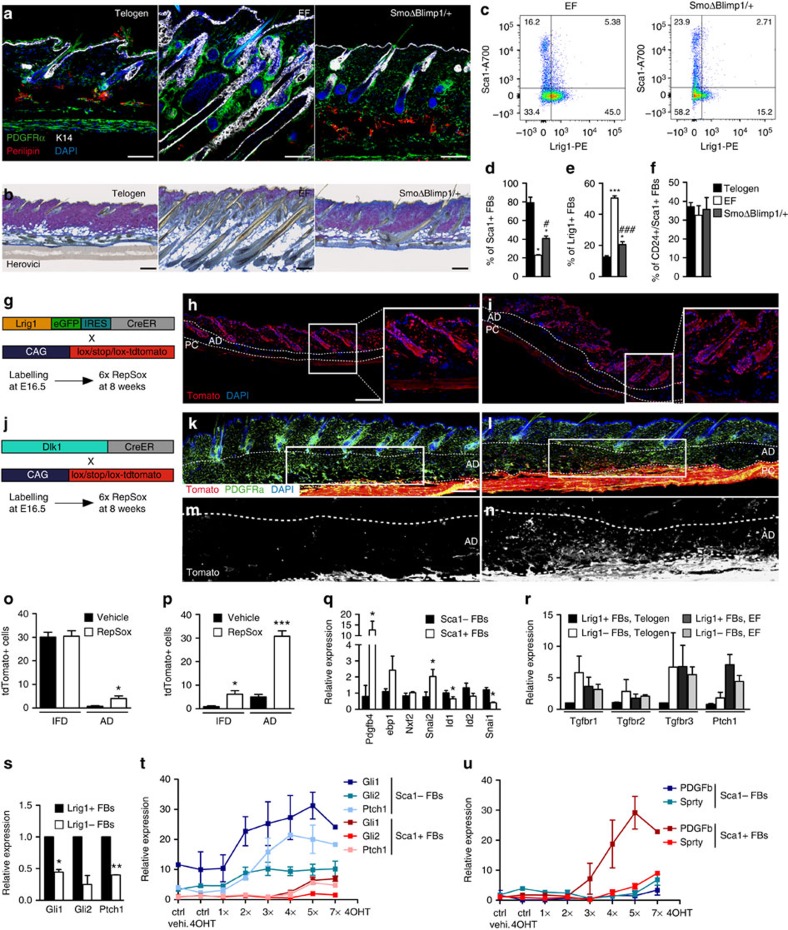
Epidermal β-catenin activation induces Hh and TGF-β signalling in distinct fibroblast lineages (**a**,**b**) Immunolabelling (**a**) and Herovici staining (**b**) of skin from *K14ΔNβ-cateninER* mice in telogen, following 4OHT treatment to induce ectopic hair follicles (EFs) or in combination with heterozygous *Smoothened* deletion via *Blimp1Cre* (*SmoΔBlimp1/+*). Scale bars, 200 μm. (**c**) Representative flow cytometry plots of Sca1+ and Lrig1+ fibroblasts after gating on PDGFRαH2BeGFP+ cells. (**d**–**f**) Summary of flow cytometric analysis. Data represent mean±s.e.m. (*n*=3). *^,#^*P*≤0.05; ***^,###^*P*≤0.0005 by a one-way analysis of variance, compared with telogen or EF skin, respectively. (**g**–**i**) Labelling Lrig1+ cells. (**g**) Experimental strategy; (**h**,**i**) skin sections from adult mice treated with vehicle (**h**) or RepSox (**i**). Insets are higher magnification images. (**j**–**n**) Labelling Dlk1+ cells. (**j**) Experimental strategy; (**k**–**n**) skin sections from adult mice treated with vehicle (**k**,**m**) or RepSox (**l**,**n**). (**m**,**n**) Grey scale images with higher magnification of the red channel in **e**,**f**, respectively. AD, adipocyte layer; PC, panniculus carnosus. Dotted lines denote thickness of adipocyte layer. Scale bars, 200 μm. (**o**,**p**) Quantification of tdTomato+ cells within the interfollicular dermis (IFD) and AD of skin treated with RepSox or vehicle in *Lrig1CreER* (**o**) or *Dlk1CreER* (**p**) transgenic mice shown in **g**–**n**. Data represent mean±s.e.m. (*n*=3; 3 fields quantified per biological sample). *P≤0.05, ***P≤0.0005 by Student's *t*-test. (**q**–**s**) Relative expression of TGF-β (**q**) and Hh target genes (**s**) and TGF-β receptors (TGFBR; **r**) in Sca1− and Sca1+ (**q**) or Lrig1+ and Lrig1− (**r**,**s**) fibroblasts in adult dermis. Data represent mean±s.e.m. (*n*=3–4). Data were normalized to Sca1− or Lrig1+ cells in telogen. *P≤0.05, **P≤0.005 by Student's *t*-test. (**t**,**u**) Relative expression of Hh target genes (**t**) and TGF-β and FGF target genes (**u**) in Sca1+ and Sca1− fibroblasts isolated from *K14ΔNβ-cateninER/PDGFRαH2BeGFP* double transgenic mice treated with 4OHT once or up to seven times. Data represent mean±s.e.m. (*n*=2; pooled from 3 to 5 mice).

Our data indicate that paracrine Hh signalling primarily stimulates papillary fibroblast proliferation, which is required for ectopic HF formation, whereas TGF-β selectively inhibits proliferation of the lower dermal lineage and stimulates adipocyte differentiation and ECM remodelling. To investigate whether, as predicted, selective inhibition of Hh in papillary fibroblasts had the same effect as inhibition in total dermal fibroblasts, we deleted one *Smoothened* allele via *Blimp1Cre*^7^ and crossed the mice with *K14ΔNβ-cateninER* mice (*SmoΔBlimp1/+*; Fig. 6a–f). As in the case of *Smoothened* deletion via *Dermo1Cre* or *PDGFRα-CreER*, *SmoΔBlimp1/+* skin harboured fewer fibroblasts in the papillary and reticular dermis and dermal sheath on epidermal β-catenin activation than controls (Fig. 6a,b and Supplementary Fig. 5n). *SmoΔBlimp1/+* skin had a reduced ability to enter anagen or form EFs and the proportions of Lrig1+, Sca1+ and Sca1+/CD24+ fibroblasts were consistent with telogen (Fig. 5c–f), with fewer Lrig1+ fibroblasts. Although the number of pre-adipocytes was not affected by *Smo* deletion (Fig. 6f), *K14ΔNβ-cateninER SmoΔBlimp1/+* skin harboured fewer mature adipocytes (Fig. 6a,b and Supplementary Fig. 5o), and ECM remodelling was reduced (Fig. 6b).

To confirm our prediction that TGF-β2 mediates its effects on the lower dermis, we performed lineage tracing experiments in combination with RepSox treatment (Fig. 6g–n). We crossed *Lrig1CreER* or *Dlk1-CreER* mice with *LSL-tdTomato* mice, then labelled the different lineages by injecting tamoxifen at E16.5 (Fig. 6g,j). When we treated the mice with RepSox at the age of 8 weeks (Fig. 6g–n), we did not detect any difference in the distribution or abundance of Lrig1+ cells (Fig. 6h,i,o), except for the appearance of labelled adipocytes (Fig. 6o). However, RepSox induced proliferation of tdTomato-positive reticular fibroblasts, which remained largely confined to the lower dermis (Fig. 6k–n), and led to a substantial increase in tdTomato+ cells in the adipocyte layer (Fig. 6p). Thus, our findings indicate that TGF-β signalling affects the lower fibroblast lineage.

### Differential expression of TGF-β and Hh target genes

Collectively, our studies show that different fibroblast subpopulations respond differently to TGF-β and Hh, with papillary, Lrig1+, fibroblasts primarily mediating the Hh response and lower dermal fibroblasts and adipocytes mediating the TGF-β response. This led us to examine whether different fibroblast subpopulations differ in expression of Hh and Tgf-β target genes (Fig. 6q–s). Consistent with our model, Lrig1+ fibroblasts expressed higher levels of Hh target genes (Fig. 6s). Sca1+ fibroblasts expressed higher TGF-β target genes such as *PDGFβ* and *Snai2*, and lower levels of *Id1* and *Snai1*, which are known to be downregulated in tumour-promoting conditions^29^,^30^ (Fig. 6q). We also found that TGF-β receptors (TGFBR) were expressed at higher levels in Lrig1− fibroblasts compared with Lrig1+ cells in adult telogen skin (Fig. 6r), although in skin with ectopic follicles there was no difference between Lrig1+ and Lrig1− fibroblasts (Fig. 6r).

qRT–PCR analysis of Hh target gene expression in flow-sorted PDGFRαH2BeGFP+/Sca1− or PDGFRαH2BeGFP+/Sca1+ fibroblasts from *K14ΔNβ-cateninER* skin revealed that after two doses of 4OHT Hh target genes were elevated to a much greater extent in Sca1− than Sca1+ fibroblasts (Fig. 6t). Hh target genes were induced in reticular fibroblasts only after four doses of 4OHT and at a much lower level than in papillary fibroblasts (Fig. 6t). In contrast, the TGF-β target gene *PDGFb* was upregulated maximally after five doses of 4OHT and to a much greater extent in Sca1+ than Sca1− fibroblasts (Fig. 6u). The FGF target gene *Sprouty* was induced with slower kinetics than Hh and TGF-β target genes. In addition, it was expressed at similar levels in Sca1+ and Sca1− fibroblasts (Fig. 6u).

From this analysis, we can conclude that not only are Hh and TGF-β target genes induced with different kinetics in response to epidermal β-catenin activation (Fig. 2d), but in addition, the induction of Hh genes is primarily attributable to Lrig1+ upper dermal fibroblasts, whereas the TGF-β response is mediated by Sca1+ lower dermal fibroblasts. As Shh stimulates fibroblast proliferation, whereas TGF-β does not (Fig. 2), this would account for the increase in the proportion of papillary fibroblasts in response to epidermal β-catenin. The stimulation of Sca1+ fibroblast proliferation is likely attributable, at least in part, to FGF signalling.

## Discussion

We have shown that the remodelling of adult dermis that occurs in response to sustained epidermal Wnt/β-catenin activation is mediated by secreted signals with differential effects on different fibroblast subpopulations. TGF-β signalling acts primarily on the lower fibroblast lineage, which corresponds to the Dlk1 lineage at E16.5 and the cells that express Sca1 in adult skin. In contrast, Shh stimulates proliferation of Sca1-/Lrig1+ fibroblasts, which in adult skin are found throughout the papillary and reticular dermis but are particularly associated with the HFs.

It has recently been demonstrated that fibroblasts originating from cells that are engrailed-1 (En-1) positive during early embryogenesis are responsible for the bulk of connective tissue deposition in dorsal skin^31^, a function that is attributed to reticular fibroblasts. These cells are located primarily in the upper dermis during embryonic development and are scattered throughout the interfollicular dermis in adult skin. In telogen skin, these cells, comprising 70% of total fibroblasts, express CD26, whereas 95% of fibroblasts that are not derived from En-1+ embryonic cells are CD26−. Our flow cytometric analysis is consistent with these observations. We found that 70–80% of fibroblasts from adult telogen skin co-expressed Sca1 and CD26 and thus correspond to the En-1+ reticular lineage (Fig. 4j and Supplementary Fig. 4b), with the remaining cells being primarily Sca1−Lrig1+. While Lrig1+ cells were more widely distributed in the dermis than previously reported, they were particularly abundant in the dermal sheath, consistent with their embryonic origin in the papillary dermis.

In the skin, the Hh pathway has long been known to be crucial for maintaining the epidermal stem cell population, and regulating the development of HF and sebaceous glands^25^. Our studies demonstrate that Hh signalling is also important within the dermis, particularly within Lrig1+ fibroblasts. This is consistent with the known role of Hh in controlling the properties of fibroblasts in various organs. In the gut epithelium, Hh proteins act as paracrine mitogens to promote the expansion of adjacent mesenchymal progenitors^32^, and paracrine Hh signalling induces renal fibrosis^33^. Shh can act as both a short-range, contact-dependent, factor and as a long-range, diffusible morphogen^34^,^35^. LacZ-Reporter assays revealed *Gli1* and *Gli2* expression in cells of the upper dermis of telogen and anagen skin, and also in cells accumulating along anagen HF^36^. Furthermore, epidermal Shh has been shown to play a crucial role in the clustering of mesenchymal cells during HF development^37^, and in *Shh* knock-out mice, dermal condensates fail to evolve into DP^38^. This suggests paracrine signalling between epidermal and mesenchymal cells over a short range, and underpins our findings that Hh signalling is more abundant in fibroblasts with embryonic origin in the papillary dermis.

In fibroblasts, TGF-β is a potent stimulus for migration, proliferation as well as for the synthesis of ECM proteins and collagen maturation into a highly cross-linked dense matrix^39^. Thus, TGF-β signalling has crucial roles in fibrosis^40^,^41^. Nevertheless, blocking this pathway with RepSox stimulated proliferation of the reticular fibroblast lineage, and inhibited fibroblast differentiation into adipocytes. This could be explained by the fact that epidermal Wnt activation induces expression of TGF-β2, which has been shown to inhibit fibroblast proliferation in contrast to TGF-β1 (ref. 15). Furthermore, Repsox appears to induce ECM synthesis and/or inhibit collagen maturation as we found increased levels of immature collagens. One paradox that remains to be explained is why reticular fibroblasts proliferate in response to epidermal Wnt/β-catenin activation, yet are inhibited by TGF-β2. This could potentially be due to upregulation of FGF2, which stimulates fibroblast proliferation and is required for EF formation. In contrast to TGF-β2, FGF2 inhibits fibroblast collagen synthesis and downregulates genes involved in ECM remodelling^42^. Indirect evidence for a selective effect of FGF2 on reticular fibroblast proliferation comes from the observation that FGFR inhibition upregulates Hh target gene expression. It is also likely that other secreted factors contribute to dermal remodelling, including Wnt3a and Wnt5a^6^, and factors that induce Wnt signalling in the fibroblasts themselves are known to profoundly alter dermal composition^43^,^44^,^45^. Furthermore, adipocyte-derived GFs such as Platelet-derived growth factor (PDGF), which are known to control epidermal stem cell activity and regulate HF cycling^46^, could be involved, because epidermal Wnt/β-catenin activation affects the composition of the adipocyte layer, and the block in adipocyte differentiation caused by RepSox led to an increase in pre-adipocytes.

We have shown that upon epidermal Wnt activation Hh signalling is activated early in fibroblasts, whereas TGF-βR and FGFR pathways are induced later. This is in accordance with a delayed expression of TGF-β2 and FGF2 by epidermal cells, but could also be due to cross-talk between the papillary and reticular fibroblast lineages. Although it has been reported that active TGF-β signalling induces the Hh pathway^47^, our data suggest that Hh signalling is not affected in skin when mice are treated with a TGF-βR inhibitor, and vice versa. Nevertheless, when Hh signalling is inhibited, the lower dermis does not respond to epidermal β-catenin activation, suggesting that there is inter-dependence within the lineages, and a possible cross-talk between fibroblast subpopulations. Whether this is promoted by intermixing fibroblast populations due to epidermal β-catenin-mediated anagen induction remains to be explored.

The fact that epidermal Wnt activation triggers distinct signalling pathways in different fibroblast lineages is interesting in light of the well-established role of Wnt in different types of cancer including those of HFs. Shh has been shown to play a role in tumour stroma^48^, and interestingly depletion of fibroblasts can exacerbate tumour growth^49^,^50^, indicating that not all cancer stroma is tumour supportive. More focus should be placed on the roles of distinct fibroblast lineages in cancer development and progression, and possibly other diseases.

## Methods

### Mice

All animal procedures were subject to local institutional ethical approval and performed under a UK Government Home Office license. *K14ΔNβ-cateninER*^2^, *Lrig1-GFP-IRES-CreER*^20^ (*Lrig1CreER*) and *Dlk1-CreERt*^7^ (*Dlk1CreER*), and *Pdgfrα-CreER*^28^ transgenic mice were generated previously. Conditional *Smoothened* (*Smo*^*tm2Amc*^*/J*)^26^, *Dermo1Cre* (*B6.129X1-Twist2*^*tm1.1(cre)Dor*^*/J*)^27^, *Blimp1-Cre*^51^, *PdgfrαH2BeGFP*^52^ and *ROSA26-tdTomato*^21^ mice were purchased from the Jackson Laboratory. Three micrograms of 4OHT (Sigma-Aldrich) in 200 μl of acetone (vehicle) was administered topically to the back skin of adult mice to activate the *K14ΔNβ-cateninER* transgene or to delete *Smoothened*. When mice received more than one dose of 4OHT, the doses were applied every second day. Telogen samples were obtained from wild-type mice that had received the same number of 4OHT treatments as the corresponding *K14ΔNβ-cateninER*^2^ in each experiment. For the lineage tracing experiments, *Lrig1CreER* or *Dlk1CreER* transgenic pregnant females received a dose of 25 or 75 μg per g mouse of tamoxifen (Sigma-Aldrich) intraperitoneally, respectively. In some experiments, mice received a dose of 500 μg EdU (Invitrogen) dissolved in PBS intraperitoneally 3–6 h before tissues were harvested.

Mice received a dose of 1 μmol RepSox (Tocris) or 25 mg per kg body weight PD173074 dissolved in acetone (Tocris) or vehicle only topically onto the back skin. Experiments involving IPI4182 (compound 32)^16^ were performed at the Cancer Research Institute Cambridge, Cancer Research UK (CRUK). A 10 mg ml^−1^ IPI4182 stock solution was prepared in 10% (2-hydroxylpropyl)-β-cyclodextrin/0.1 M sodium citrate/phosphate, pH3 (vehicle) and administered by oral gavage at a dose of 60 mg per kg body weight per day.

No statistical methods (power calculations) were used to predetermine sample size. However, based on prior experience with the experimental models, the experiments were designed to use the smallest number of mice predicted to generate statistically meaningful data. Experiments were performed on 6- to 8-week-old sex-matched mice. Experiments were performed on male and female animals. After genotyping, mice were assigned randomly to treatment groups. Because of the severe skin phenotype of *K14ΔNβ-cateninER*^2^ mice treated repeatedly with 4OHT, blinded assessment of experimental outcomes was not always applicable; however, it was used whenever possible. Mice were excluded from analysis only if they had to be euthanized because of health concerns.

### Histology and microscopy

Paraffin- or optimal cutting temperature compound-embedded tissues were sectioned and stained^6^,^7^ using the following primary antibodies (all diluted 1:100 unless stated otherwise) for immunofluorescence labelling: Lrig: R&D Systems, FAB3688G; CD26: R&D Systems, AF954; Sca1: R&D Systems, AF1226; PDGFRa: R&D Systems, AF1062; Collagen III: Abcam, ab7778, Collagen11a1: Abcam, ab64883; Elastin: Abcam, ab21610; Caveolin: Cell Signaling Technology, 3267; phospho-Histone H3 (Ser10) antibody: Cell Signalling Technology, 970; Active Caspase-3: RnD Systems, AF835; K14: Covance, PRB-155P, 1:500; GFP: Abcam, ab13970, 1:500; RFP: Rockland, 600-401-379, 1:300. EdU staining was performed with a Click-it EdU imaging kit (Invitrogen) according to the manufacturer's recommendations. Images were acquired with a Nikon A1 Upright Confocal microscope. Images of H&E- and Herovici-stained sections were acquired with a Hamamatsu slide scanner. Representative images of skin from two to three independent experiments with at least three biological replicates per group are shown.

### Flow cytometry and cell sorting

Dermal cells for fluorescence-activated cytometric cell sorting and analysis^6^,^7^ were labelled according to standard procedures with the following antibodies: anti-ITGA6-APC (AbSource, 1:20), Lrig1-PE (RnD systems, 1:20), anti-CD24-PE clone: M1/69 (BioLegend, 1:100), anti-Ly-6A/E (Sca1)-BV605 (BD Horizon, 1:200) or Alexa Fluor 700, anti-CD26-APC and anti-CD140a-PE or APC (eBioscience, 1:20). Click-it EdU Flow Cytometry Assay Kits (Invitrogen) were used to detect proliferating cells. Labelled cells were analysed on a BD LSRFortessa cell analyser. 4,6-Diamidino-2-phenylindole or LIVE/DEAD Fixable Violet Dead Cell Stain (Life Technologies) was used to exclude dead cells.

### Cell culture

Mouse primary fibroblasts were isolated^6^ and cultured in DMEM supplemented with 10% FCS or Amniomax (Gibco). Cells were pulsed with 10 μM EdU 3–4 h before fixation and staining with the Click-it EdU Imaging Kit (Invitrogen). To stimulate or inhibit specific signalling pathways in fibroblasts, the following concentrations of GFs and inhibitors were added to the medium for 24 or 48 h: Shh 1 μg ml^−1^; TGF-β2 10 ng ml^−1^; FGF2 20 ng ml^−1^; FGF5 10 ng ml^−1^ (all from RnD Systems; vehicle: PBS); IPI4182 0.5 μM (Infinity Pharmaceuticals; vehicle: DMSO); RepSox 25 μM (Tocris; vehicle: DMSO); PD173074 2 μM (Tocris; vehicle: DMSO). DED was prepared by floating 5 mm diameter punch biopsies of back skin on 0.8% trypsin (Gibco) dissolved in PBS for 60 min, separating the dermis from the epidermis with forceps and depleting cells from the tissue by at least ten freeze–thaw cycles. Before fibroblasts were seeded onto DEDs, the tissue was placed into 24-well cell culture inserts and equilibrated with medium.

### qRT–PCR

Total RNA was purified with the Purelink RNA micro kit (Invitrogen) with on-column DNaseI digestion, according to the manufacturer's instructions. RNA was reverse transcribed with SuperScriptIII (Invitrogen). PCR reactions were performed with TaqMan Fast Universal PCR Master Mix and Taqman probes purchased from Invitrogen.

### Graphing and statistical analysis

All graphs were generated using Excel and GraphPad Prism 6 software. Data are means±standard error of the mean (s.e.m.). One-way analysis of variance parametric test with Bonferroni Post-Test or Student's *t*-tests were performed for experiments, with *P*<0.05 considered significant.

## Additional information

**How to cite this article:** Lichtenberger, B. M. *et al*. Epidermal β-catenin activation remodels the dermis via paracrine signalling to distinct fibroblast lineages. *Nat. Commun.* 7:10537 doi: 10.1038/ncomms10537 (2016).

## Supplementary Material

Supplementary Information Supplementary Figures 1-5
