# Supplementary material for: Epidermal β-catenin activation remodels the dermis via paracrine signalling to distinct fibroblast lineages
Source: Nat Commun. 2016 Feb 3;7:10537. doi: 10.1038/ncomms10537 (PMC4742837; doi:10.1038/ncomms10537)
Supplement: Supplementary Information — Supplementary Figures 1-5 [file ncomms10537-s1.pdf]

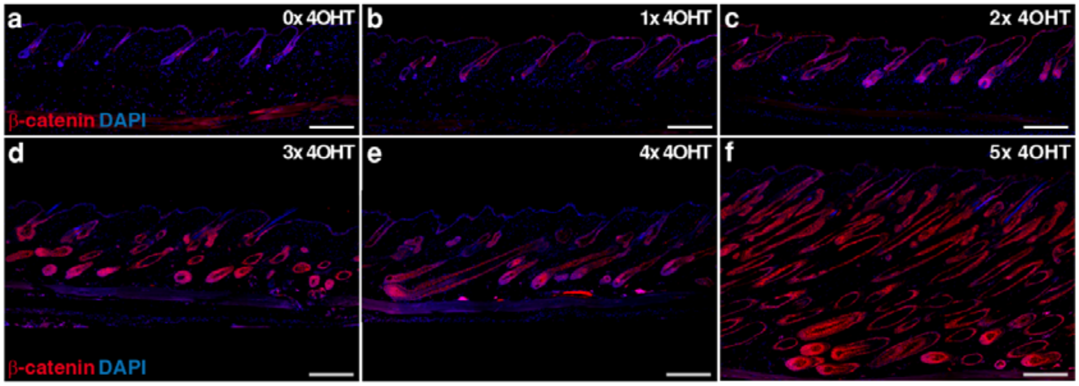

Supplementary Figure 1: Sustained tamoxifen-induced β-catenin stabilisation in the epidermis induces ectopic follicle formation. Skin sections from *K14ΔNβ-cateninER/ PDGFRaH2BeGFP* double transgenic mice treated up to 5 times with 4OHT were stained with an antibody detecting Non-phospho (active) β-Catenin (Ser33/37/Thr41; Cell Signaling Technology, #8814). DAPI was used to detect nuclei. Scale bars represent 200 μm.

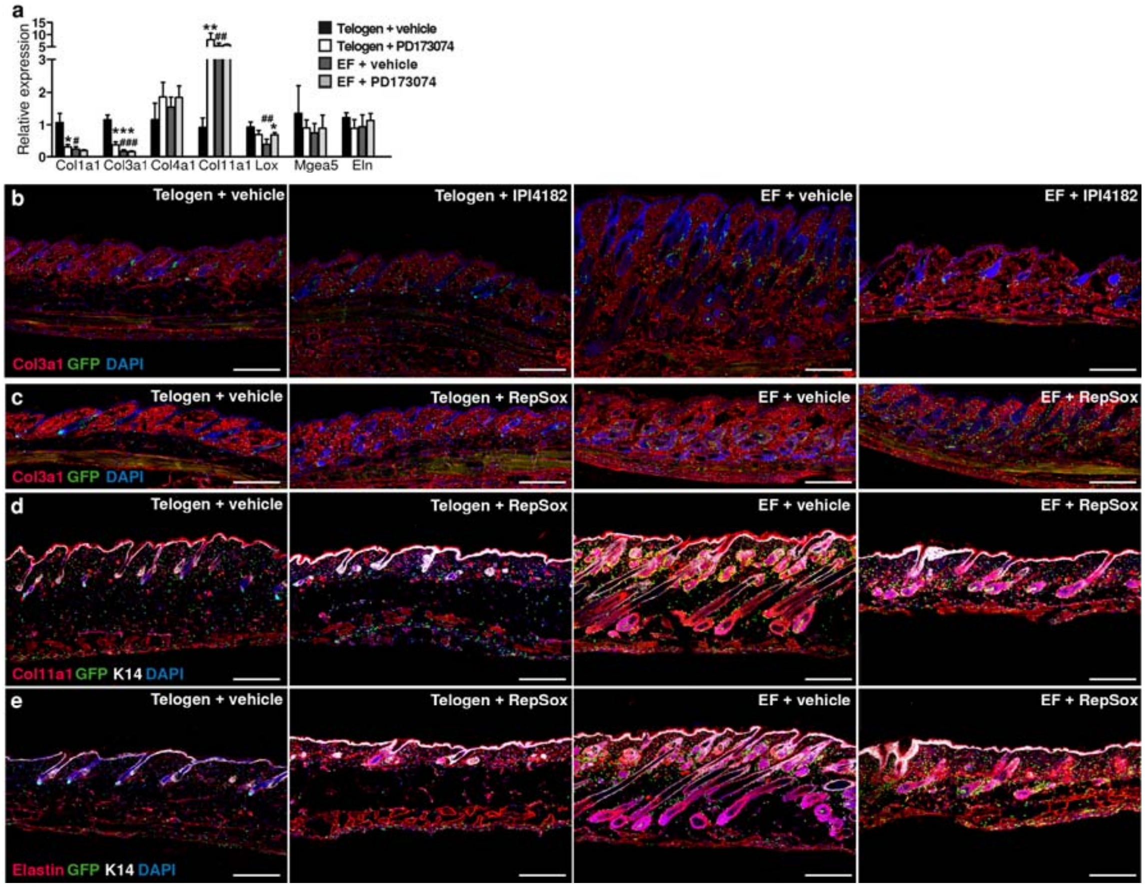

Supplementary Figure 2: Effects of IPI4182, RepSox and PD173074 on ECM remodelling. (a) Bar graph showing relative expression of the indicated genes in fibroblasts isolated from telogen and reprogrammed skin treated with PD173074 or vehicle. Data represent mean ± SEM (n=4-5). * p≤0.05; ** p≤0.005; *** p≤0.0005, inhibitor-treated compared to vehicle-treated mice; # p≤0.05, ## p≤0.005; ### p≤0.0005, compared to telogen skin, by a one-way ANOVA. **(b-e)** Representative images of skin sections immunolabelled with antibodies to Collagen type 3, Collagen type 11, Elastin, K14 and GFP, counterstained with DAPI. Scale bars represent 200 µm.

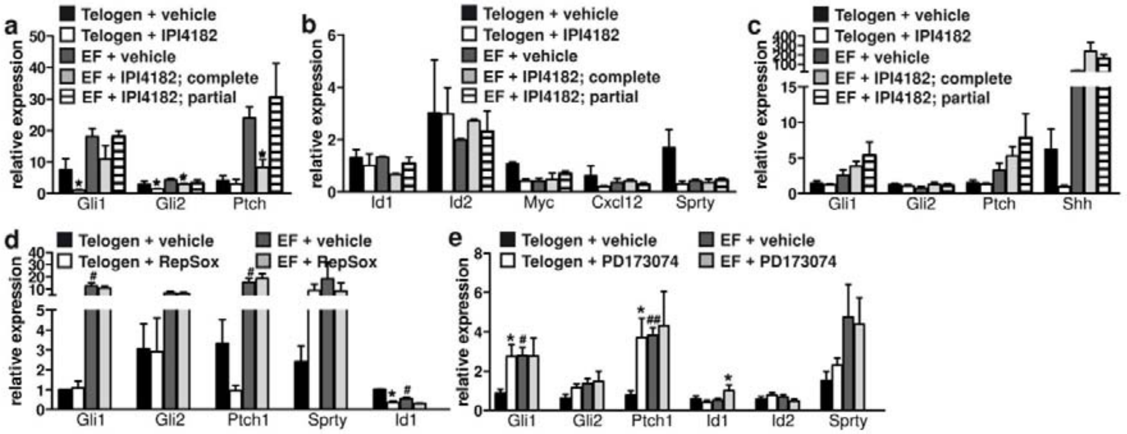

Supplementary Figure 3: Effects of pharmacological inhibitors on Hh, TGFβ and FGF target gene expression. **(a-c)** Bar graphs showing relative expression of the indicated genes in fibroblasts (a, b) and epidermal cells (c) isolated from telogen and reprogrammed skin treated with the Hh inhibitor IPI4182 or vehicle. **(d, e)** Bar graph showing relative expression of the indicated genes in fibroblasts isolated from telogen and reprogrammed skin treated with the TGFβ inhibitor RepSox (d) or the FGFR inhibitor PD173074 (e) or vehicle. Data represent mean ± SEM (n=4-5). * $p \leq 0.05$, inhibitor-treated compared to vehicle-treated mice; # $p \leq 0.05$ and ## $p \leq 0.005$, EF skin compared to telogen skin.

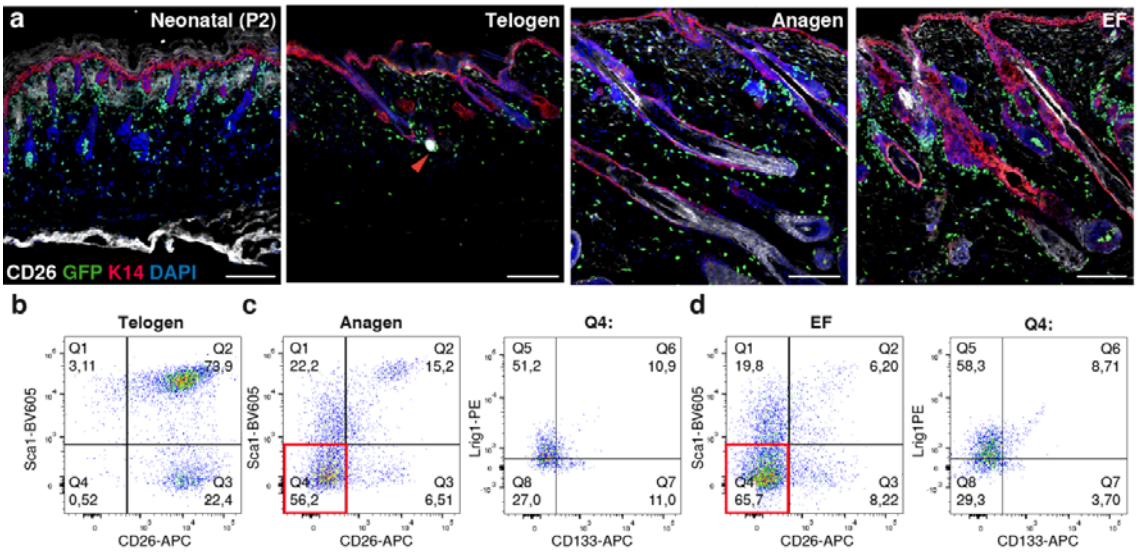

Supplementary Figure 4: Changes in dermal CD26 and CD133 expression in response to epidermal β-catenin activation. (**a**) Sections from neonatal, adult telogen, anagen and reprogrammed skin (EF) of *K14ΔNβ-cateninER/ PDGFRaH2BeGFP* double transgenic mice stained with the indicated antibodies. DAPI was used to detect nuclei. Scale bars represent 200 µm. Red arrow head highlights CD26+ dermal papilla cells in telogen skin. **(b-d)** Representative FACS plots showing fibroblast populations stained with antibodies detecting Sca1, CD26, Lrig1 and CD133 isolated from telogen (b), anagen (c) and reprogrammed skin after gating on PDGFRαH2BeGFP+ cells. In the right hand panels of (c, d) Sca1-/CD26- cells in quadrant 4 (left hand panels) are shown labelled for Lrig1 and CD133.

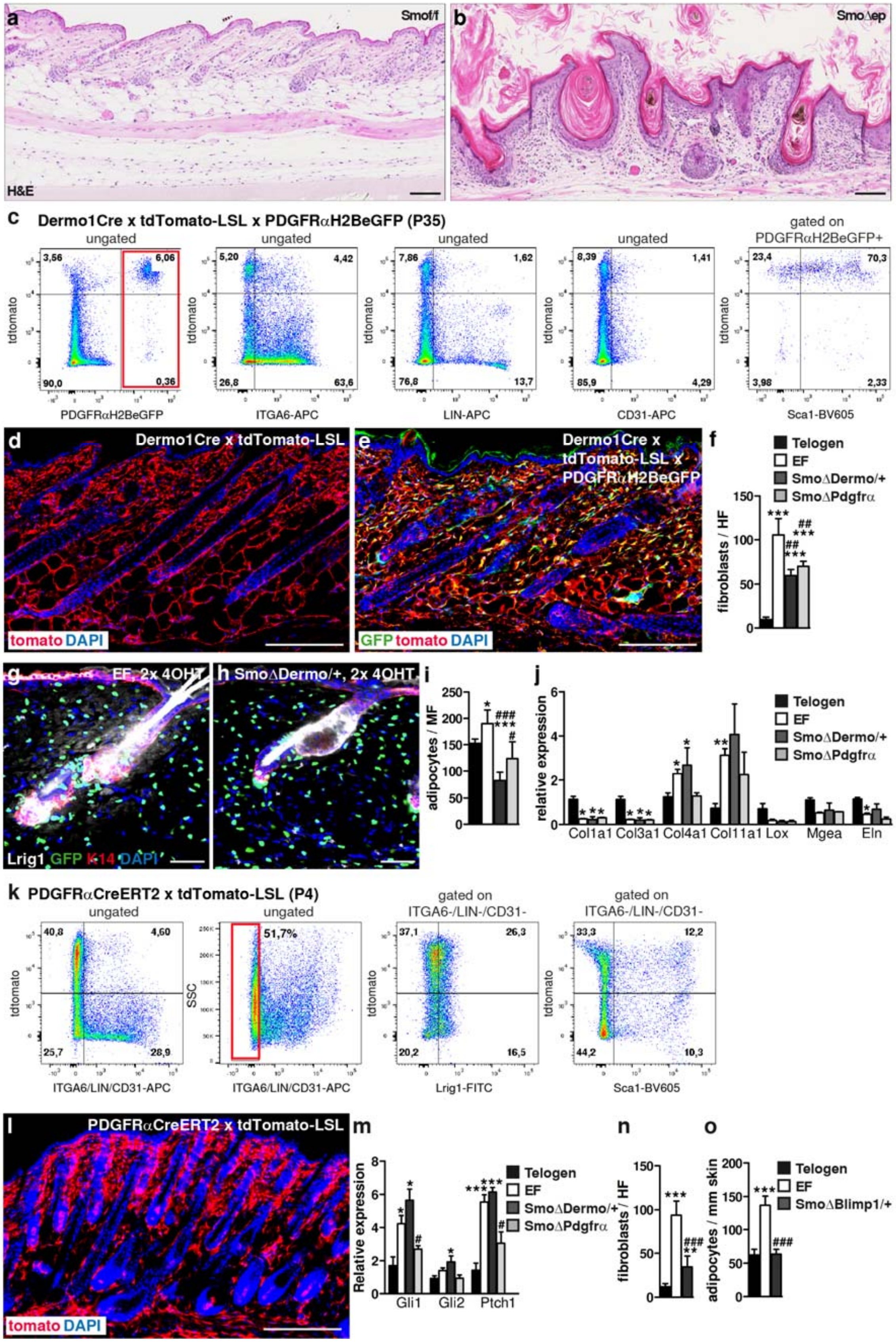

a
Smof/f
H&E
b
SmoΔep
c
Dermo1Cre x tdTomato-LSL x PDGFRαH2BeGFP (P35)
ungated
gated on PDGFRαH2BeGFP+
tdtomato
PDGFRαH2BeGFP
ITGA6-APC
LIN-APC
CD31-APC
Sca1-BV605
d
Dermo1Cre x tdTomato-LSL
tomato DAPI
e
Dermo1Cre x tdTomato-LSL x PDGFRαH2BeGFP
GFP tomato DAPI
f
Telogen
EF
SmoΔDermo/+
SmoΔPdgfrα
fibroblasts / HF
g
EF, 2x 4OHT
h
SmoΔDermo/+, 2x 4OHT
Lrig1 GFP K14 DAPI
i
adipocytes / MF
j
relative expression
Col1a1
Col3a1
Col4a1
Col11a1
Lox
Mgea
Eln
k
PDGFRαCreERT2 x tdTomato-LSL (P4)
gated on ITGA6-/LIN-/CD31-
ITGA6/LIN/CD31-APC
SSC
Lrig1-FITC
l
PDGFRαCreERT2 x tdTomato-LSL
m
Relative expression
Gli1
Gli2
Ptch1
n
o
adipocytes / mm skin
SmoΔBlimp1/+

Supplementary Figure 5: Skin phenotype of mice lacking epidermal or dermal *Smoothened*. (a,b) H&E stained back skin sections of a 20 day-old *Smo∆ep* mouse lacking epidermal *Smoothened* (b) and the littermate control (a). Scale bars represent 200 µm. **(c)** Representative FACS plots showing that the *Dermo1Cre* transgenic line efficiently targets both Sca1+ and Sca1- fibroblasts. Percentages of targeted epidermal cells (ITGA6+), haematopoietic cells (LIN+) and endothelial cells (CD31+) are also indicated. **(d, e)** Immunofluorescent staining of skin sections with antibodies to RFP and GFP, counterstained with DAPI, showing that the *Dermo1Cre* transgenic line efficiently targets a high proportion of fibroblasts in all regions of the dermis. Scale bars represent 200 µm. **(f)** Number of fibroblasts surrounding the permanent portions of HF quantified in immunostained skin sections from the indicated genotypes. Data represent mean ± SEM (n=6; 5 HF were quantified per biological sample). *** p≤0.0005 compared to telogen skin. ## p≤0.005; ### p≤0.0005 compared to EF skin, by a one-way ANOVA. **(g, h)** Immunofluorescent staining with antibodies detecting Lrig1, GFP and K14 of skin sections from *K14ΔNβ-cateninER* (g) and *K14ΔNβ-cateninER Smo∆Dermo/+* mice (h) after three 4OHT treatments. Scale bars represent 100 µm. **(i)** Number of adipocytes per microscopic field quantified in H&E sections. Data represent mean ± SEM (n=3). * p≤0.05; *** p≤0.0005 compared to telogen skin. # p≤0.05; ### p≤0.0005 compared to EF skin, by a one-way ANOVA. **(j)** Bar graph showing relative expression of the indicated genes in fibroblasts isolated from back skin of mice with the indicated genotypes. Data represent mean ± SEM (n=3-5). * p≤0.05, ** p≤0.005, compared to telogen skin, by a one-way ANOVA. **(k)** Representative FACS plots showing that the *PDGFRαCreERT2* transgenic line efficiently targets both Lrig1+ and Sca1+ fibroblasts. Percentages of targeted epidermal cells (ITGA6+), haematopoietic cells (LIN+) and endothelial cells (CD31+) are also indicated. **(l)** Immunofluorescent staining of skin section with antibodies to RFP, counterstained with DAPI, showing that the *PDGFRαCreERT2* transgenic line efficiently targets fibroblasts throughout the dermis. Scale bars represent 200 µm. **(m)** Relative expression of the indicated genes in fibroblasts isolated from telogen, EF, *Smo∆Dermo/+* or *Smo∆Pdgfrα* mice. Data represent mean ± SEM (n=3-5). * p≤0.05; *** p≤0.0005 compared to telogen skin. # p≤0.05 compared to EF skin, by a one-way ANOVA. **(n)** Number of fibroblasts surrounding the permanent portions of HF quantified in immunostained skin sections from the indicated genotypes. Data represent mean ± SEM (n=3; 5 HF were quantified per biological sample). ** p≤0.005; *** p≤0.0005 compared to telogen skin. ### p≤0.0005 compared to EF skin, by a one-way ANOVA. **(o)** Number of adipocytes per mm skin quantified in H&E sections. Data represent mean ± SEM (n=3). *** p≤0.0005 compared to telogen skin. ### p≤0.0005 compared to EF skin, by a one-way ANOVA.
